# Three‐Dimensional Heterogeneous Bonding for High‐Density and Low‐Noise TMR Sensing Arrays

**DOI:** 10.1002/advs.202520686

**Published:** 2025-12-26

**Authors:** Zi'ang Han, Zhenhu Jin, Chenglong Zhang, Jiamin Chen

**Affiliations:** ^1^ State Key Laboratory of Transducer Technology Aerospace Information Research Institute Chinese Academy of Sciences Beijing China; ^2^ School of Electronic Electrical and Communication Engineering University of Chinese Academy of Sciences Beijing China; ^3^ College of Materials Sciences and Opto‐Electronic Technology University of Chinese Academy of Sciences Beijing China

**Keywords:** high‐density magnetic sensing arrays, low‐frequency 1/f noise suppression, magnetoresistance enhancement, three‐dimensional heterogeneous integration bonding, tunneling magnetoresistance sensors, wafer‐level bonding and etching optimization

## Abstract

Tunneling magnetoresistance (TMR) devices are crucial for high‐density low‐power magnetic sensing owing to the increase in data‐intensive applications. This study presents a three‐dimensional heterogeneous integration bonding technique for TMR sensors. The number of serially connected magnetic tunnel junctions is doubled without enlarging the chip area by vertically bonding two TMR film stacks, and the low‐frequency 1/f noise is effectively suppressed. The sputtering parameters of the Cr/Au bonding layer, argon‐ion activation conditions, and bonding pressure are optimized to achieve a void ratio of only 0.73%. Double‐layer TMR devices are fabricated via backside silicon removal, photolithography, and ion‐beam etching. A 45° etching angle mitigates sidewall redeposition, increases the magnetoresistance ratio from 149% to 172%, and decreases the magnetic noise to 0.97 nT·Hz^−1/2^ at 1 Hz. The proposed approach enables high integration and low noise without altering the device dimensions and has strong potential for applications in high‐density magnetic‐sensing arrays. The three‐dimensional heterogeneous bonding strategy proposed in this study vertically integrates double‐layer TMR films, doubles the number of tunnel junctions without enlarging the device footprint, and significantly suppresses the 1/f noise through an optimized bonding process. This approach enables high‐density, low‐noise, low parasitic resistance magnetic sensing for next‐generation spintronic devices.

## Graphical abstract

This study demonstrates a three‐dimensional heterogeneous bonding approach to fabricate compact TMR sensing units with double junction numbers and improved magnetic performance. Optimized Au─Au bonding and angled etching improve device integrity, noise characteristics, and magnetoresistance. This method provides a scalable route to dense, low‐noise spintronic sensor arrays suitable for emerging data‐intensive applications.

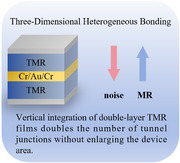

## 1 Introduction

The rapid development of data‐intensive applications ranging from autonomous vehicles to implantable medical devices [1] has imposed unprecedented demands on magnetic sensing technologies: devices must achieve low‐power operation and high sensitivity [2] with minimal noise [3], while maintaining compact form factors. Among various candidates, tunneling magnetoresistance (TMR) [4] and giant magnetoresistance (GMR) [5] multilayers have emerged as core technologies for high‐performance magnetic sensing, owing to their high magnetoresistance (MR) ratios and excellent magneto‐electric conversion efficiency [6]. However, conventional lateral lithographic scaling has approached its physical limits [7]. Although reducing the size of magnetic tunnel junctions (MTJs) can increase the series resistance and, to some extent, increase sensitivity [8], it simultaneously results in a sharp increase in process complexity, higher parasitic resistance, and amplified thermal noise, which ultimately degrades the signal‐to‐noise ratio [9]. Therefore, novel paradigms that transcend the lateral scaling framework are essential to achieve optimal sensitivity and noise performance while maintaining controllable device dimensions.

Three‐dimensional (3D) heterogeneous integration bonding [10], wherein functional magnetic layers are vertically bonded instead of relying on lateral patterning, is an attractive route for device integration. Yakushiji et al. [11] recently demonstrated room‐temperature Ta–Ta direct bonding to bond p‐MTJ multilayer films with a lead electrode layer based on low‐resistance CuN, combined with backside silicon removal. This enabled damage‐free transfer of ultrathin perpendicularly magnetized MTJ nanopillars onto complementary metal–oxide–semiconductor wafers. The bonded interfaces were free of voids and their magnetic properties remained intact. They achieved defect‐free bonding by employing an atomically flat Ta capping layer. The subsequent silicon removal step preserved the spin‐transfer torque switching behavior and the MR ratio of the MTJs. Chen et al. [12] further extended this 3D concept to room‐temperature Au–Au direct bonding of current perpendicular to plane giant magnetoresistance (CPP‐GMR) multilayer films and polycrystalline multilayer bottom electrodes based on half‐metallic Heusler alloys. They designed suitable seed and buffer layers to induce (001)‐oriented epitaxial growth on silicon substrates and suppress the detrimental diffusion of aluminum from NiAl into the Heusler electrodes during high‐temperature annealing. The MR ratio of the device after integration was comparable to those of single‐crystalline MgO references, highlighting the universality of 3D heterogeneous integration bonding across different spintronic materials.

The core innovation of this study lies in the proposal and experimental validation of a 3D heterogeneous integration bonding technique tailored for TMR sensors. Our primary objective is to double the number of effective serially connected MTJs without increasing the chip footprint. Consequently, we systematically reduce the parasitic resistance and ultimately achieve simultaneous noise suppression and MR ratio enhancement. Our approach significantly improves the integration density and magnetic field resolution of TMR sensors and offers a scalable manufacturing pathway for high‐density, low‐noise, low parasitic resistance magnetic sensing arrays.

The experimental section focuses primarily on the 3D heterogeneous integration bonding technique. During the bonding of the TMR film stacks, key steps, such as the sputtering of the bonding layer and backside silicon removal were systematically optimized, with particular attention paid to mitigating the impact of the interface roughness on the bonding quality. Subsequently, the magnetic moments of the films were characterized using a vibrating‐sample magnetometer (VSM) to elucidate the influence of the bonding process on the magnetic properties of the films. In the device fabrication stage, double‐layer TMR structures were realized via photolithography and etching, and the MR ratio, magnetic noise, and sensitivity at different etching angles were systematically evaluated. The results demonstrate that this bonding technique effectively reduces low‐frequency 1/f noise while maintaining the planar dimensions of the devices, thereby providing a novel pathway for the development of highly integrated and high‐performance magnetic sensors.

## 2 Experimental

### 2.1 3D Heterogeneous Integration Bonding Process

Customized multilayer TMR films were used in this study. The stack structure of the films was Si/SiO_2_/Ta(6)/Ru(14)/Ta(6)/ Ru(14)/Ta(6)/Ru(5)/Pt_75_Mn_25_(24)/Co_75_Fe_25_(2)/Ru(0.65)/CoFeB(2)/ MgO(2.5)/CoFeB(3)/NiFe(20)/IrMn(12)/Ta(6)/Ru(10) (units in nm). The 8‐inch TMR wafers were diced into 3 cm × 3 cm chips using a dicing saw. Nanometallic films of 5 nm Cr/15 nm Au were deposited via magnetron sputtering as interlayer dielectric films for surface‐activated bonding (SAB) [13, 14]. During SAB, the Cr layer not only acted as an adhesion layer between Au and the metal substrate but also effectively prevented interdiffusion between Ru and Au. Subsequently, the Au surface was activated via argon‐ion treatment to form dangling metal bonds [15]. Finally, the samples were transferred to a wafer bonder for room‐temperature bonding.

SAB at room temperature requires an extremely low surface roughness. Therefore, the sputtering parameters of the bonding layer must be investigated to optimize the deposition process and achieve minimal roughness. The surface of the sputtered bonding layer was treated with Ar ion activation to further improve the bonding performance. Subsequently, the activated samples were bonded using an EVG wafer bonder. The bonded samples were examined using a scanning acoustic microscope (SAM), and the acquired images were analyzed to evaluate the bonding quality.

**FIGURE 1:**
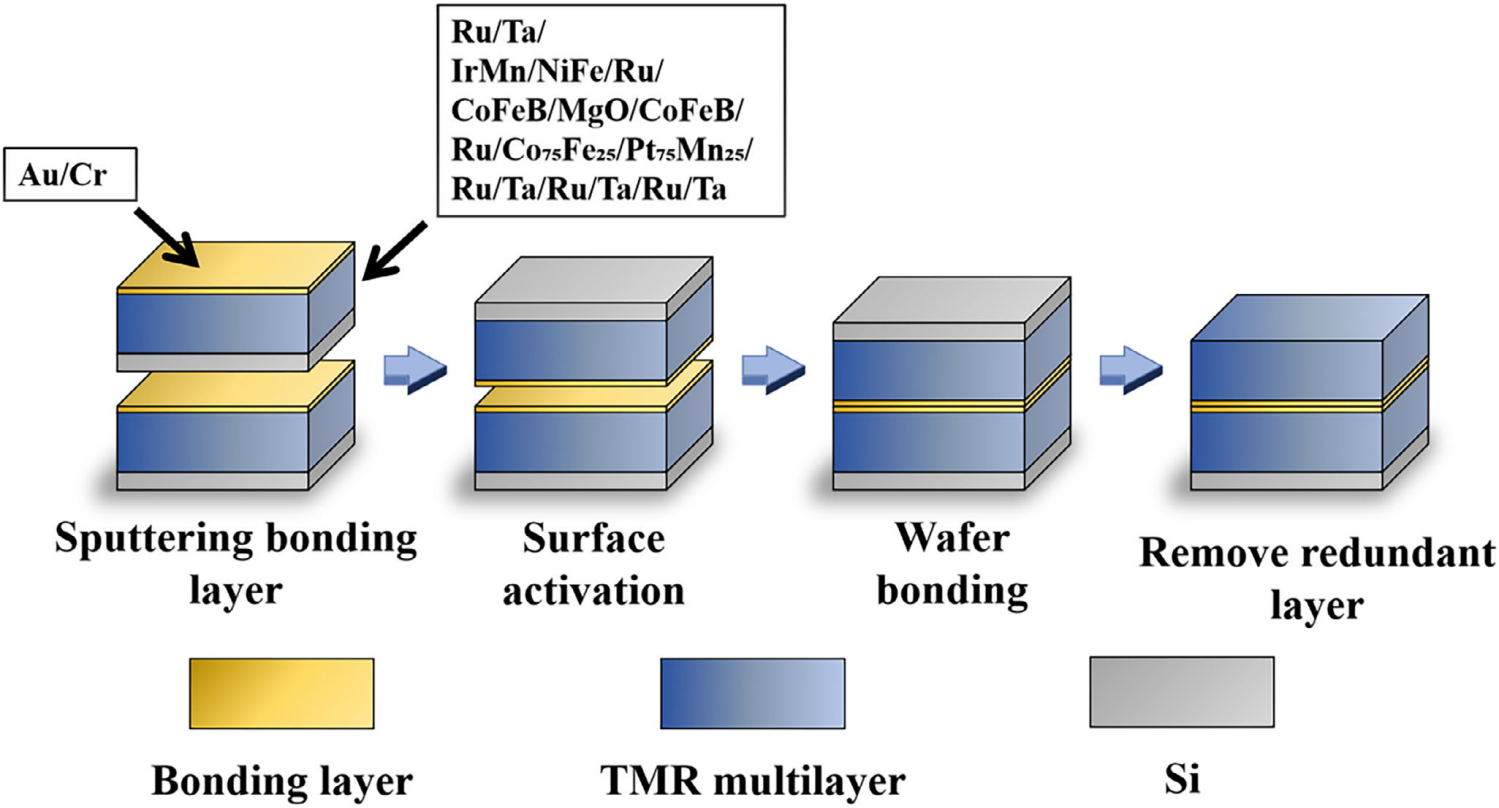
Schematic of the 3D heterogeneous bonding process.

The top and bottom surfaces of the bonded sample are Si substrates, and the presence of Si significantly slows down the etching process and reduces precision during patterning. Therefore, the first step in the fabrication of double‐layer TMR devices is the removal of one side of the Si substrate. First, thin Si substrates were subjected to chemical mechanical polishing (CMP). Thinning was performed on a cast iron plate using Al abrasives with a particle size of 9 µm. When the substrate thickness was reduced to approximately 200 µm, coarse polishing was performed on a polishing pad using Al abrasives with a particle size of 1 µm for approximately 1 h. Once a mirror‐like finish was achieved on the Si surface, coarse polishing was stopped, and a polishing slurry was used for fine polishing to further reduce the surface roughness. The Si substrate remaining after CMP was removed via deep reactive ion etching (DRIE) until the SiO_2_ layer was exposed on the top surface. Subsequently, reactive ion etching (RIE) was employed to remove the 1 µm‐thick SiO_2_ layer and expose the Ta seed layer at the surface. A schematic of the 3D heterogeneous integration bonding process is shown in Figure 1.

### 2.2 Fabrication of Double‐Layer TMR Devices

Following the backside Si removal process, the RIE‐treated sample surface was bombarded using an oxygen ion etcher to remove the by‐products generated during RIE [16]. The surface resistance, measured thereafter using a multimeter, was approximately 10 Ω. This result confirmed that the Ta layer beneath the SiO_2_ was exposed at the surface and that the by‐products were completely eliminated, enabling the fabrication of series‐connected MTJ arrays.

A four‐step photolithography process—exposure, etching, passivation layer deposition, and Au electrode sputtering—was employed to fabricate serial MTJ arrays. The photoresists used for exposure were SPR 330 and AZ 5214, and the developer was NMD‐3. The serial MTJ arrays were patterned via ion‐beam etching (IBE), and endpoint detection was monitored using a spectrometer.

**FIGURE 2:**
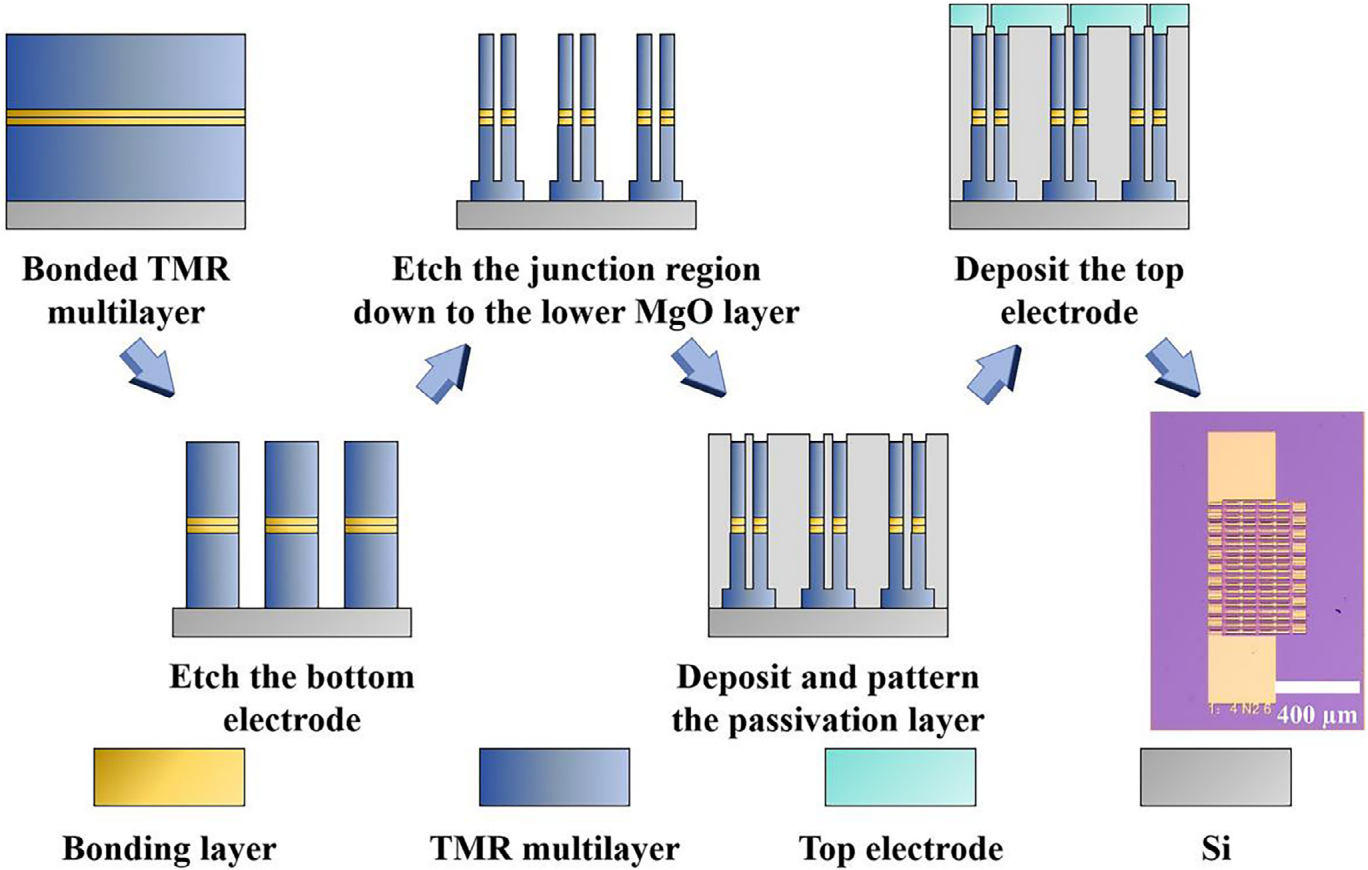
Fabrication of the MTJ array after wafer bonding of the TMR multilayer stack.

In the first step, IBE was applied to define the bottom electrodes of the MTJs by etching down to the SiO_2_ layer at the bottom of the stack. In the second step, the free layer was patterned with the etching endpoint set at the MgO layer to minimize the parasitic resistance without affecting the tunneling effect. The third step involved the deposition of a passivation layer. SiN was deposited via inductively coupled plasma chemical vapor deposition (ICP‐CVD), followed by RIE to open windows in the SiN and expose the MTJ areas. In the final step, Au electrodes were deposited across the entire chip surface via magnetron sputtering, and excess Au was removed via a lift‐off process. Figure 2 shows the fabrication process and an optical image of the MTJ array.

The performance of the series‐connected MTJ arrays was characterized. First, the MR curves were measured via the four‐probe method. The resistances in the parallel and antiparallel states of the arrays were obtained from MR curves. Subsequently, a magnetic noise measurement system was used to evaluate the noise and sensitivity of the MTJ arrays.

## 3 Results and Discussion

### 3.1 Dependence of the Surface Roughness on the Sputtering Parameters and Ar Ion Activation Parameters

**FIGURE 3:**
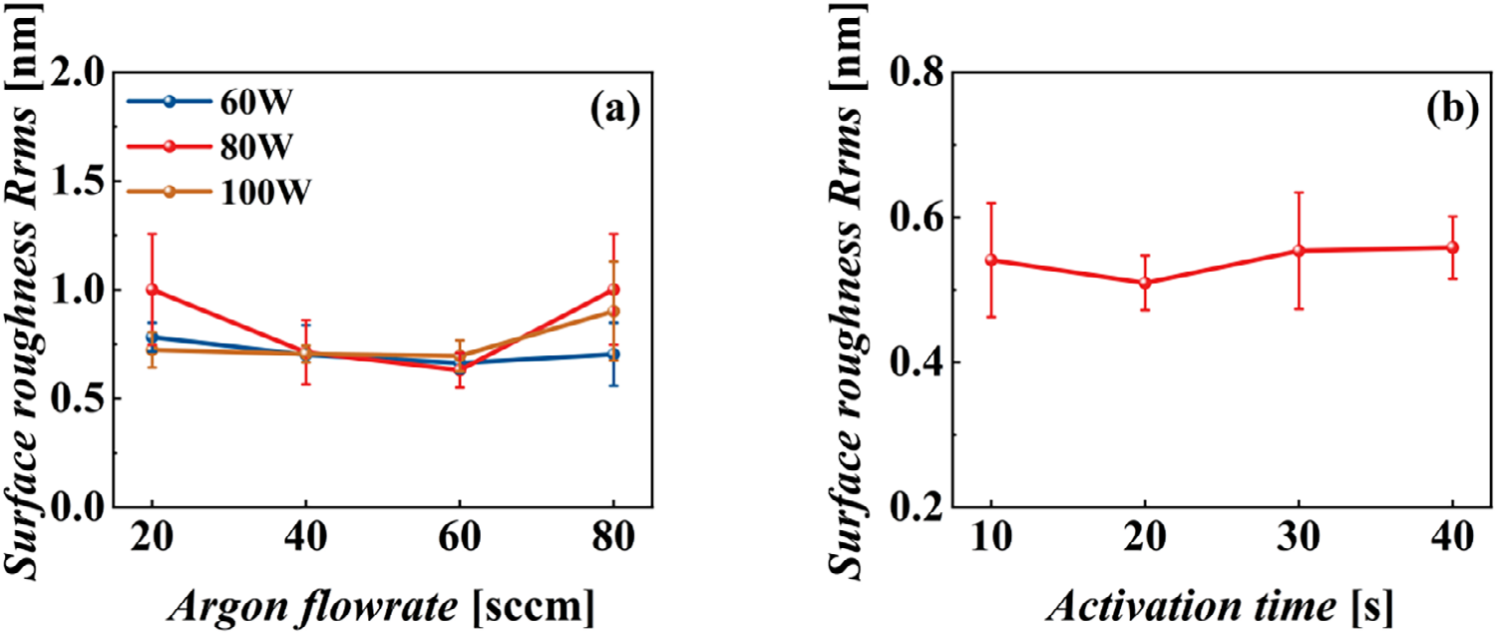
(a) Effect of Ar flow rate and sputtering power on the surface roughness of the samples deposited via magnetron sputtering. (b) Effect of different Ar ion activation times on the surface roughness of the samples. Data are presented as mean ± standard deviation (SD) (n = 15 devices per group).

A 5 nm Cr/15 nm Au bonding layer was deposited on 3 cm × 3 cm chips via magnetron sputtering. The surface roughness was minimized by tuning the Ar gas flow rate and sputtering power using a ULVAC QAM magnetron sputtering system. The Ar flow rates during Au deposition were set to 20, 40, 60, and 80 sccm, and the power was set to 60, 80, and 100 W. The resulting twelve parameter combinations were used for sputtering. The surface roughness was characterized using a white‐light interferometer. Five points (four at the edges and one at the center) were measured for each sample over a scanning area of 1 mm × 1 mm. The results, including the error bars, are shown in Figure 3a. At all power settings, the surface roughness decreased gradually with an increase in the Ar flow rate, and reached a minimum at 60 sccm. Excessively high Ar flow can increase the chamber pressure and lead to frequent collisions between sputtered target atoms before reaching the substrate, which reduces their kinetic energy and degrades directionality. Conversely, sustaining the glow discharge in the chamber at an excessively low Ar flow is challenging and leads to unstable sputtering rates. Both conditions result in increased surface roughness [17]. As shown in Figure 3a, the error bars were the smallest at an Ar flow rate of 60 sccm, indicating that the planar uniformity of the sputtered films was the best at 60 sccm. The surface roughness increased with a further increase in the Ar flow. As shown in Figure 3a, the roughness was the minimum with a root mean square (RMS) roughness of 0.631 nm at an Ar flow of 60 sccm and a sputtering power of 80 W. These optimal sputtering parameters were adopted in the subsequent experiments.

The effect of Ar ion activation time on the sample surface at the optimal bonding layer sputtering parameters was further investigated. The Ar flow was set to 200 sccm and the sputtering power was set to 200 W, with activation times of 10, 20, 30, and 40 s. The surface roughness of the samples after Ar ion bombardment was measured using a white‐light interferometer, as shown in Figure 3b. The roughness reached a minimum at 20 s, with an average RMS roughness of 0.509 nm. This reduction is primarily attributed to the initial removal of surface contaminants and the planarization of microprotrusions, resulting in a smoother surface. With an increase in the activation time, excessive sputtering gradually introduced local micro‐pits and differential etching, leading to an increase in the surface roughness [18]. However, at 30–40 s, sputtering and redeposition reached a dynamic balance, and the roughness stabilized. Overall, compared to the pre‐activation state, the surface roughness decreased by approximately 0.122 nm and was favorable for subsequent wafer bonding.

### 3.2 Bonding Quality Dependence of Bonding Pressure

The effect of the bonding pressure on the bonding quality was investigated. The films were first treated at optimal sputtering and Ar ion activation parameters, and the activated samples were immediately transferred to the bonder to prevent surface contamination. The bonding chamber was evacuated to an ultra‐high vacuum of 8 × 10^−^⁴ mbar, and bonding pressures of 1000, 1500, 2000, 2500, and 3000 N were applied with a bonding time of 60 s. The bonded samples were maintained at room temperature under atmospheric pressure for 24 h. No interfacial fractures were observed, indicating good bonding quality.

**FIGURE 4:**
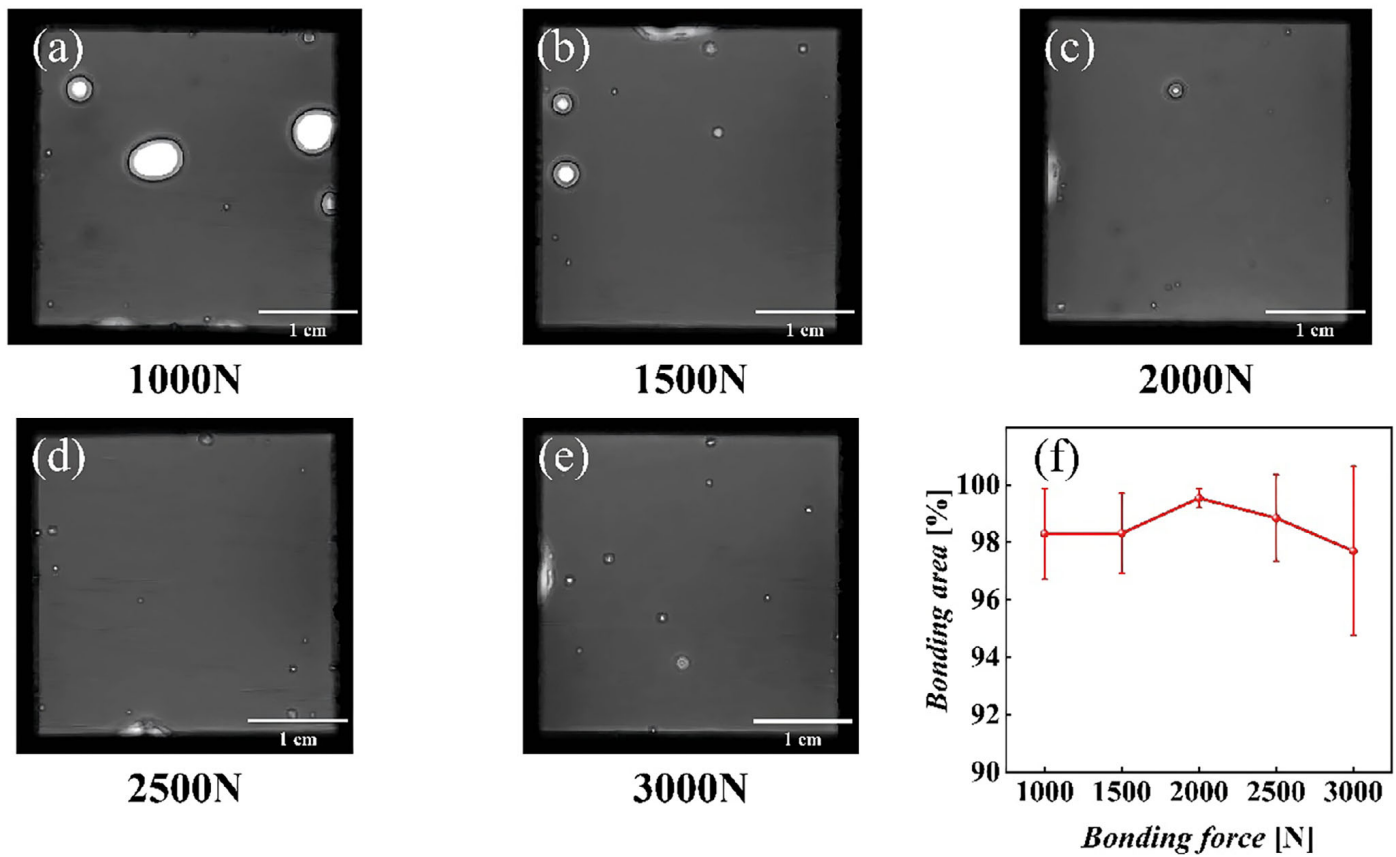
SAM images of the samples bonded under different bonding pressures. (a‐e) 1000, 1500, 2000, 2500, and 3000 N, respectively. (f) Relationship between the bonding pressure and bonded area ratio. Data are presented as mean ± SD (n = 15 devices per group).

The bonded samples were examined using an SAM. Figure 4 shows a plot of the bonded area at different bonding pressures along with error bars. The bonding quality was the best at 2000 N, with a void rate of only 0.73%. When the bonding pressure was below 2000 N, bonding was incomplete because of the insufficient van der Waals contact area due to surface nonuniformity. Conversely, at bonding pressures >2000 N, the excessive pressure introduced defects, such as dislocation pile‐ups and microcracks, at the bonding interface, leading to a rapid decrease in the bonded area. The corresponding results are shown in Figure 4. The white regions indicate the small surface protrusions that caused partial bonding failure.

### 3.3 Magnetic Characterization of Films via Vibrating Sample Magnetometry

**FIGURE 5:**
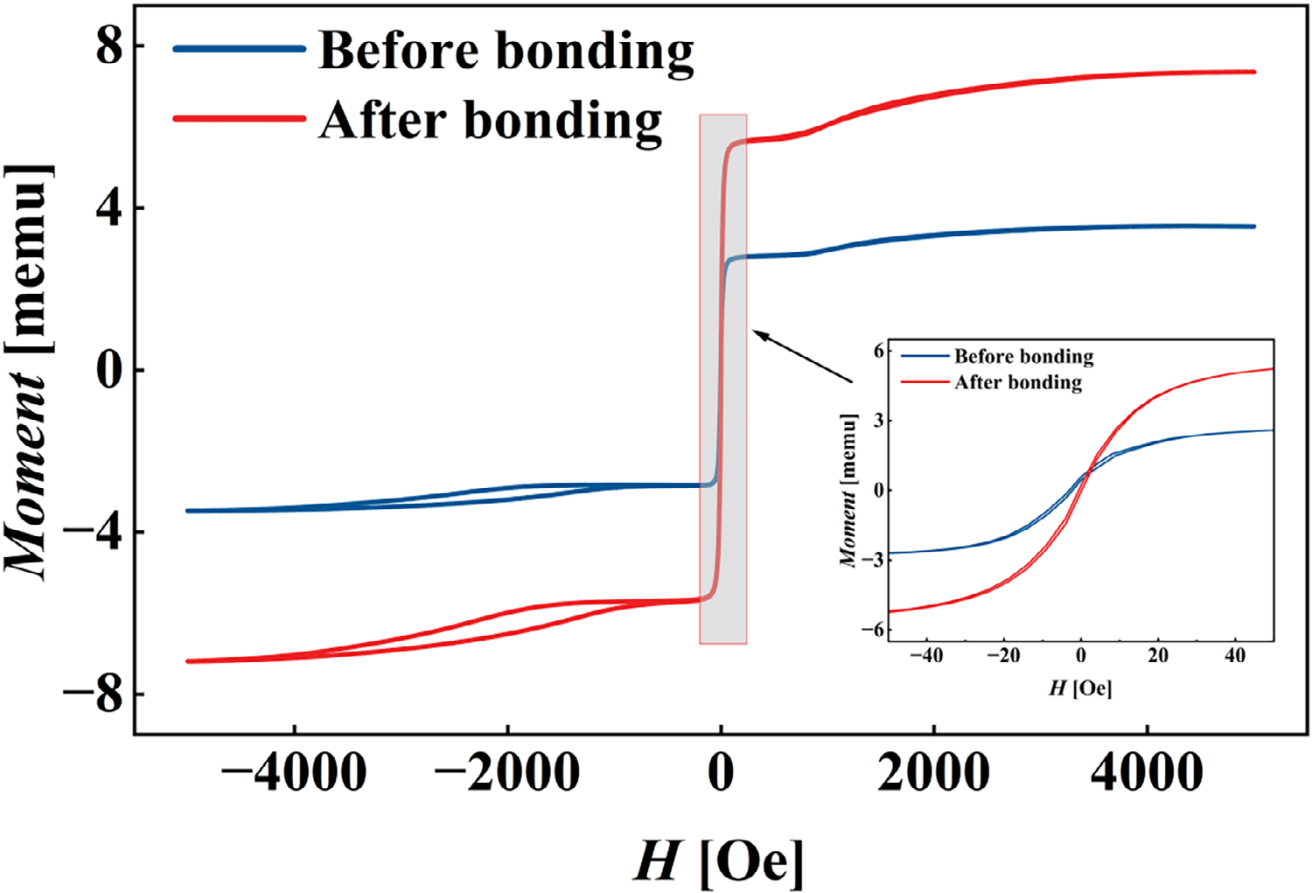
Results of VSM measurements of the TMR film stacks before and after bonding.

The TMR effect, which is the core principle of TMR devices, relies on spin‐polarized electrons tunneling through an insulating barrier layer. Therefore, the ferromagnetic properties of TMR stacks are of critical importance. Potential damage to the magnetic layers (due to bonding) that could affect the TMR performance was investigated via VSM measurements. The applied magnetic field was oriented in‐plane, with a field range of ± 5000 Oe. The results are shown in Figure 5. Under an applied field of 5000 Oe, the magnetic moments of the free, reference, and pinned layers were all aligned along the field direction. As the strength of the external magnetic field decreased, the reference layer first underwent magnetization reversal. However, the magnetic moment of the free layer remained aligned with the external field owing to the effect of the top‐pinned layer. Magnetization reversal in the free layer was observed when the external field strength was further decreased to nearly zero. With an increase in the applied field strength to –5000 Oe, the magnetization of the bottom pinned layer aligned with the external field. The overall hysteresis loops before and after bonding exhibited essentially identical trends. The magnetic moment of the bonded chip was approximately twice that of the unbonded chip. The coercive field changed from *H_c_* = 1.43 × 10^−^⁴ Oe before bonding to *H_c_* = 2.46 × 10^−^⁴ Oe after bonding, corresponding to an approximately twofold increase. These results demonstrate that the bonding process does not deteriorate the TMR characteristics of the multilayer TMR stack and that the bonded films remain fully functional for device applications.

### 3.4 Magnetoresistance Analysis of Serial MTJ Arrays

**FIGURE 6:**
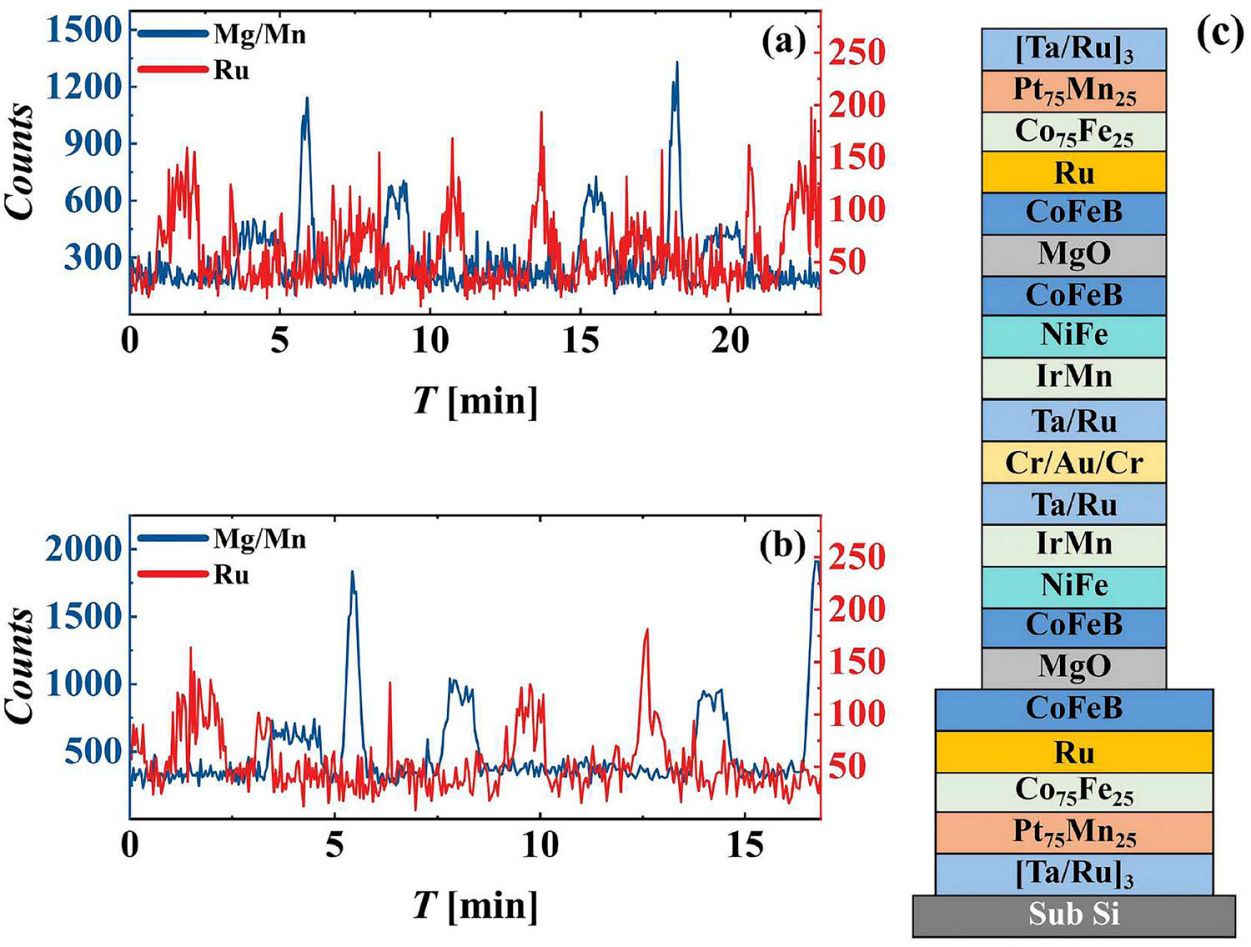
Endpoint detection spectra during the (a) first and (b) second etching steps. (c) Schematic of the multilayer structure of the TMR.

The bottom electrode in the bonded TMR films was first etched, after the removal of the backside silicon. The sample stage was inclined at 15° relative to the ion source during IBE. A second etching step was planned for sidewall cleaning, and hence, the redeposition effects were not considered during the first etching. As illustrated in the schematic of the film structure in Figure 6c, the first etching stopped upon reaching the underlying Si substrate. Endpoint monitoring was performed using spectroscopic signals. The blue curve in Figure 6a represents the spectral lines of Mg and Mn; etching reached the underlying Pt_75_Mn_25_ layer after six peaks appeared. The appearance of the Ru peak (red curve) indicated that etching had reached the bottom Si layer, and thereafter, etching was stopped.

The second etching step was performed to define the MTJ area. In single‐layer TMR devices, the second etching step typically stops at the MgO layer. For the bonded double‐layer TMR films, etching was stopped at the lower MgO layer. This approach ensures proper operation of both the upper and lower TMR devices while minimizing the influence of parasitic resistance on the overall device performance. Figure 6b shows the spectrum of the second etching step; the high‐intensity peaks in the blue curve correspond to the Mg content in the MgO layer. Etching was stopped upon the appearance of a second high peak.

**FIGURE 7:**
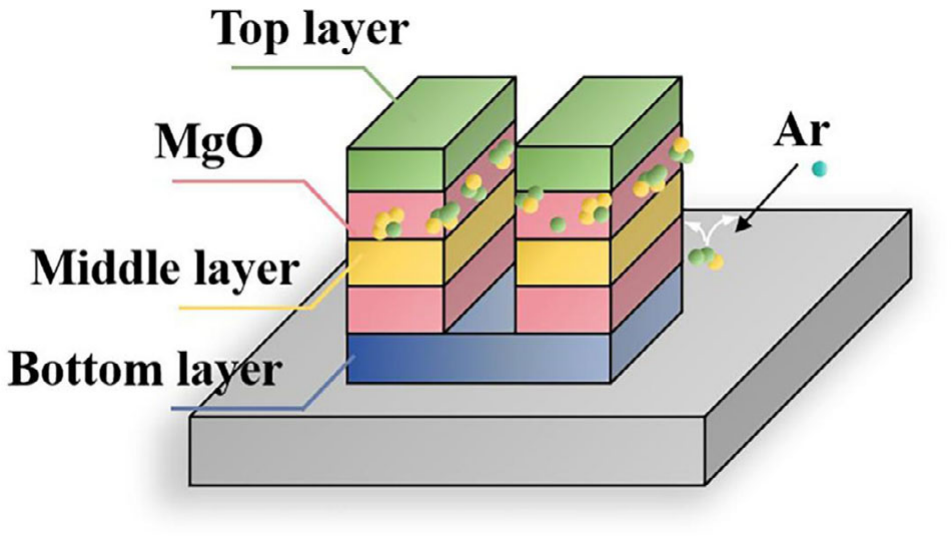
Schematic of sidewall redeposition during etching.

Four etching angles were used during the second etching step. The thickness of the sample film after bonding was approximately 400 nm. When etching reached the lower MgO layer, the upper MgO layer was encapsulated by other conductive atoms owing to redeposition. Consequently, a parallel circuit that weakens or even eliminates the effect of the upper TMR layer was formed. As shown in Figure 7, the top layer refers to the total stack of films above the upper MgO layer in the bonded TMR structure, and the bottom layer denotes the total stack of films below the lower MgO layer. A second etching step was performed at different angles to address redeposition; the stage‐to‐ion source angles were set to 15°, 30°, 45°, and 60° to investigate the influence of the etching angle on TMR device performance. The results indicated that adjusting the etching angle can effectively mitigate sidewall redeposition and related issues. The experimental results are detailed below.

Serial MTJ arrays with aspect ratios of 1:1, 1:2, 1:3, and 1:4 were fabricated with a fixed short‐side width of 20 µm. For each aspect ratio, 120 MTJs were connected in series. The MR of the fabricated double‐layer TMR devices was measured via the four‐probe method under an external magnetic field of ±200 Oe. Devices with identical aspect ratio and number of series MTJs were selected for the MR measurements.

**FIGURE 8:**
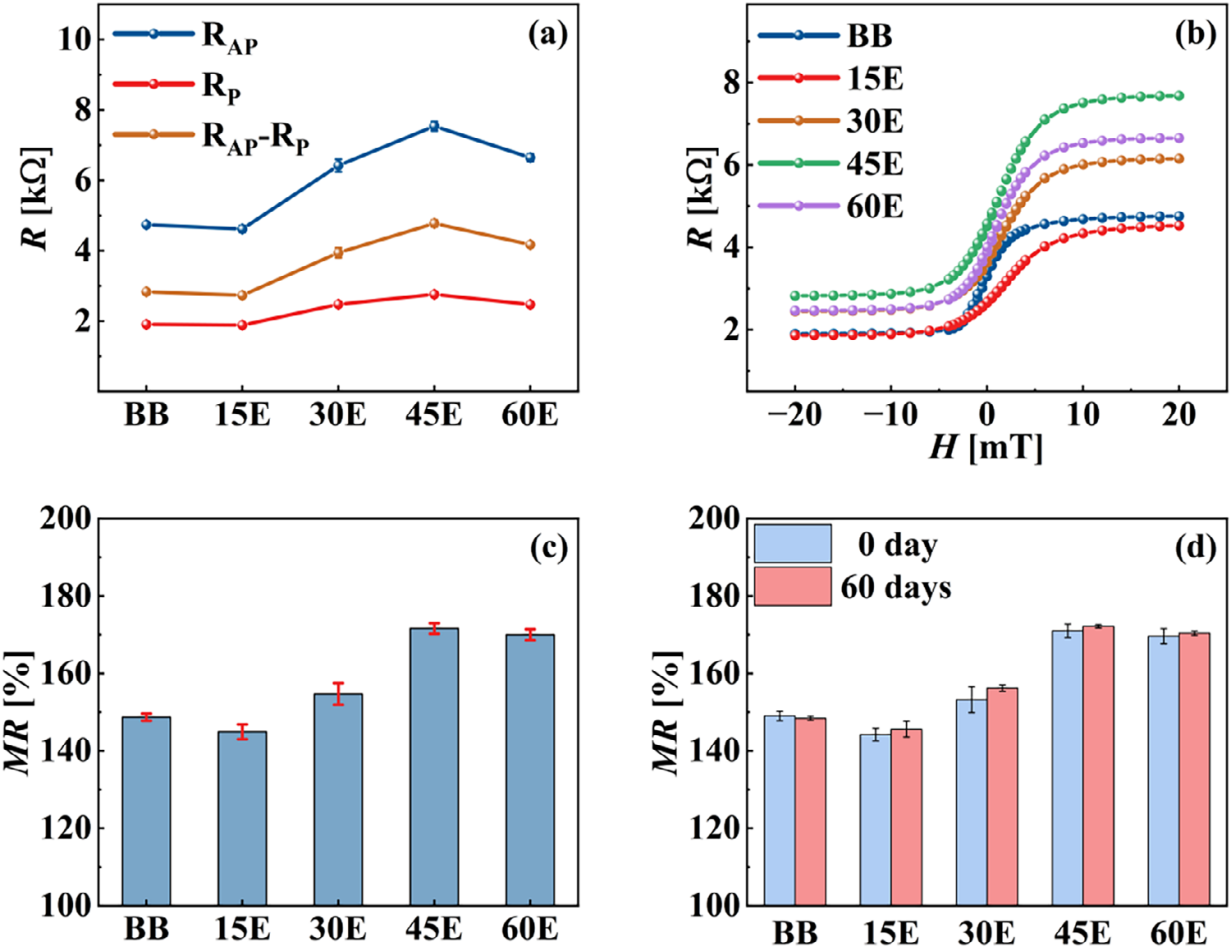
Devices measured before and after bonding at different etching angles. BB denotes the pre‐bonding state, and 15E, 30E, 45E, and 60E represent etching angles of 15°, 30°, 45°, and 60°, respectively. (a) Changes in R_P_ and R_AP_ resistances. (b) Variations in the RH curves. (c) Changes in MR values. (d) Results of MR measurements of devices at different etching angles: before bonding, after bonding, and after 60 days of storage in air. Data are presented as mean ± SD (n = 15 devices per group).

The parallel (*R_P_*) and antiparallel (*R_AP_*) resistances, as well as their difference *ΔR*, were measured before bonding, and the results are shown in Figure 8a. *ΔR* for the 15° etching angle was lower than the pre‐bonding value. This reduction was attributed to deep etching, which caused redeposition of conductive atoms near the MTJ sidewalls, leading to partial device shorting and disappearance of the TMR effect. The lateral etching rate gradually increased with an increase in the etching angle, effectively cleaning the sidewalls and enabling appropriate operation of both the upper and lower TMR devices. When the etching angle was 45°, ΔR reached its maximum value, which was approximately 1.7 times that before bonding. The final device comprised two stacked TMR multilayers, and hence, its resistance was expected to be twice that of the pre‐bonded state. The deviation from this theoretical value is attributed to parasitic resistance effects, which prevented the total resistance from reaching ideal doubling. A detailed analysis of this phenomenon is presented in the following section. However, when the etching angle increased to 60°, the resistance suddenly decreased, possibly because the horizontal etching rate in the MTJ area exceeded the vertical etching rate, damaging the MgO layer and reducing the effective resistance.

The resistance–magnetic field (RH) curves before and after bonding for the four different etching angles were plotted, as shown in Figure 8b. The shapes of the MR curves after bonding remained essentially unchanged and were consistent with the pre‐bonding RH curves. The low‐hysteresis characteristics of the double‐pinned TMR film stacks were preserved after bonding.

The MR values obtained from these measurements are shown in Figure 8c. The error bar plot shows that the MR before bonding was approximately 149%, whereas it reached a maximum of 172% after bonding at an etching angle of 45°.

After bonding, the number of MTJs is doubled from N to 2N. According to Equation (1), the measured resistances in the antiparallel and parallel states are expressed as *R_AP_* = *R'_AP_* + *R_para_* and *R_P_* = *R'_P_* + *R_para_*, respectively, where *R'_AP_* and *R'_P_* are the intrinsic MTJ resistances and *R_para_* denotes the parasitic resistance.

(1)

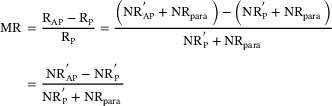




In a conventional two‐dimensional MTJ stacking scheme, doubling the number of serial MTJs would proportionally increase both the intrinsic and parasitic resistances. However, in our bonding approach, the upper and lower MTJ arrays share the same electrode after integration. Consequently, the parasitic resistance scales with N rather than 2N, Based on Equation (1), although both *R'_AP_* and *R'_P_* double due to the increased number of MTJs, the parasitic resistance remains unchanged. This asymmetry leads to an overall increase in the *MR* ratio after bonding.

The parasitic resistances of the TMR devices at different etching angles were analyzed. The resistance‐area (RA) and parasitic resistance of the devices were measured in both the *R_P_* and *R_AP_* states. The slope of the linear fit in the plots represents the RA value at each etching angle, and the intercept corresponds to the parasitic resistance. The parasitic resistance of the devices originates from the resistance of the electrodes and conductive atoms surrounding the device.

**FIGURE 9:**
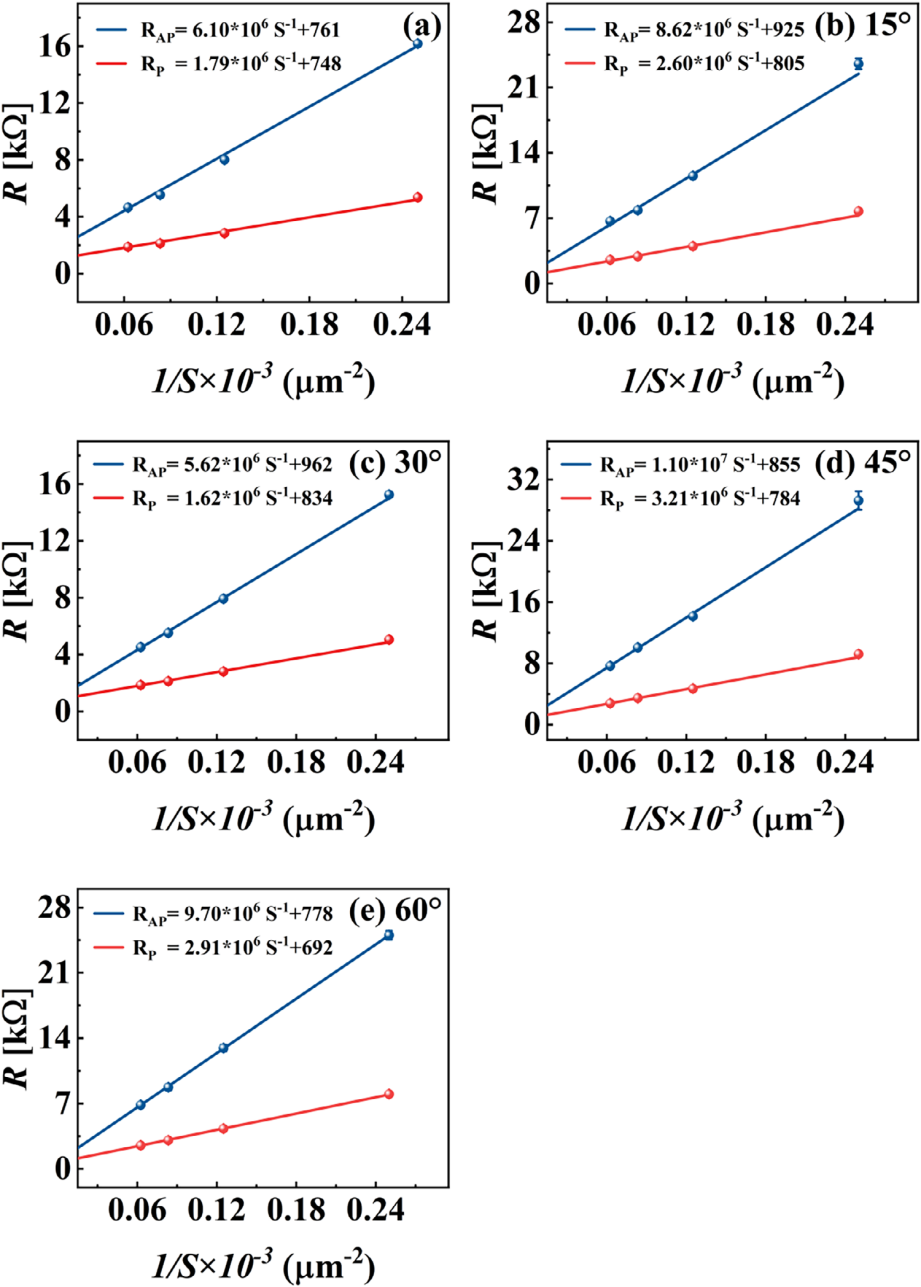
Effective junction area versus resistance for devices measured: (a) before and after bonding at different etching angles: (b) 15°, (c) 30°, (d) 45°, and (e) 60°. Data are presented as mean ± SD (n = 15 devices per group).

As shown in Figures 9b–e, the parasitic resistance decreased with an increase in the etching angle, indicating that sidewall redeposition could be effectively mitigated by adjusting the etch angle [19]. However, owing to the introduction of the bonding layer, the parasitic resistance of the bonded devices remained slightly higher (Figure 9a). The MR of the MTJs increased with increasing etching angle. At an etching angle of 15°, the parasitic resistance was higher than that at the other three angles, indicating severe redeposition of conductive atoms on the sidewalls. At 60°, the parasitic resistance reached a minimum, indicating that an increase in the etching angle can effectively help “clean” the MTJ sidewalls. However, large etching angles increase the lateral etching rate, which can damage the MgO layer in the junction area and weaken the tunneling effect.

At an etching angle of 45°, the resistance reached a value approximately 1.9 times the pre‐bonding value, which is closest to the ideal condition. This confirms that both the upper and lower TMR layers maintained the TMR effect and operated normally.

Finally, the noise and sensitivity of the fabricated devices were measured. According to Equation (2) [20], the total voltage noise spectral density of a series‐connected MTJ array comprises contributions from the 1/f noise, thermal noise, and shot noise. The 1/f component was dominant in the low‐frequency region.

(2) $$S_{V^{2}}\left(V^{2}/Hz\right)=S_{V^{2}\;,1/f}+S_{V^{2},\mathrm{therm}}+S_{V^{2},\mathrm{shot}}$$

As shown in Equation (3) [20], the 1/f noise is inversely proportional to the number of MTJs connected in series (N). In our 3D‐bonded devices, the number of series MTJs increased from R to 2R after bonding. Therefore, based on the model, a reduction of approximately 30% in the 1/f noise is expected compared to that of the pre‐bonding devices.

(3) $$S_{V,1/f}\left(f\right)\propto \frac{1}{\sqrt{N}},S_{V,shot}\left(f\right)\propto \frac{1}{\sqrt{N}}$$

**FIGURE 10:**
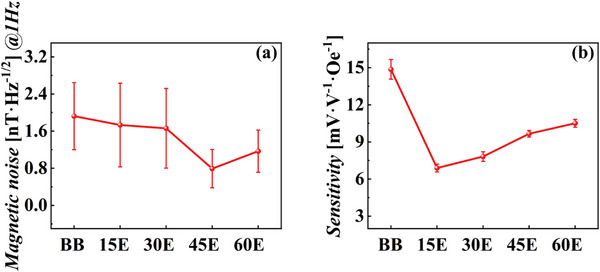
Measurement results before and after bonding under different etching angles. BB denotes the pre‐bonding state, and 15E, 30E, 45E, and 60E represent etching angles of 15°, 30°, 45°, and 60°, respectively. (a) Magnetic noise at 1 Hz. (b) Sensitivity. Data are presented as mean ± SD (n = 15 devices per group).

The experimental results shown in Figure 10a indicate that for the device fabricated with a 45° etching angle after bonding, the noise level at 1 Hz decreases from 1.92 nT (pre‐bonding) to 0.97 nT, corresponding to an overall noise reduction of approximately 50%.

Furthermore, Equation (3) predicts that the shot noise of the TMR devices decreases with an increase in the MTJ series number. Hence, the additional experimentally observed noise suppression can be attributed to the combined reduction of both the 1/f noise and shot noise in the series‐connected MTJ array.

**FIGURE 11:**
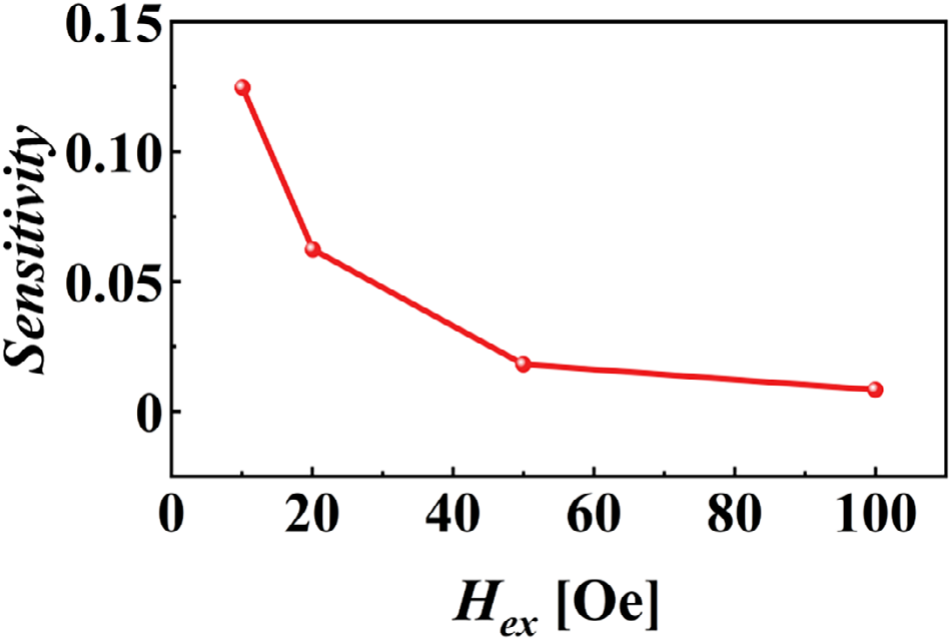
Relationship between sensitivity and exchange bias field [21].

Figure 10b shows the sensitivity curves for the TMR devices before and after bonding at different etching angles. The sensitivity of the bonded TMR devices was lower than that before bonding. This reduction was attributed to the decreased area of the pinned layer in the upper TMR device due to etching, which enhanced the effective magnetic anisotropy and consequently increased the exchange bias field (*H_ex_*). As demonstrated in previous studies, the exchange bias field is inversely related to the lateral area of the pinned layer and a lower pinned layer area leads to a stronger H_ex_. This bias field suppresses the magnetization reversal of the free layer and weakens the response of the device to an external magnetic field, resulting in lower sensitivity [21]. To further clarify this mechanism, a figure based on the referenced study was added (Figure 11), providing a more intuitive illustration of the correlation between *H_ex_* and device sensitivity.

Moreover, the increase in film thickness after bonding leads to longer etching times, which can affect the shape of the junction area and reduce the shape anisotropy field (*H_k_*). According to Equation (4) [22], a reduction in the shape anisotropy field (*H_k_*) leads to an increase in the device sensitivity.

(4) $$S=\frac{dV}{dH}\propto \frac{1}{H_{k}}$$

This is consistent with the observed variations in sensitivity of the bonded devices at the four different etching angles (Figure 10b).

The performance advantages of the proposed 3D heterogeneously bonded double‐layer TMR device were further evaluated as follows. A quantitative comparison was conducted against several representative state‐of‐the‐art commercial TMR sensor chips, including MultiDimension Technology (MDT)’s TMR 2103 [23], as well as Multi‐innovation Technology (MIT)’s Magic 2001 [24]. Table 1 summarizes the sensitivity and low‐frequency noise characteristics of these benchmark devices along with those of the device developed in this study.

**TABLE 1:** Comparison between the performance of the proposed 3D‐bonded double‐layer TMR device and commercial TMR sensor chips.

Sensor model	Sensitivity (mV·V^−1^·Oe^−1^)	Noise@1 Hz (nT·Hz^−1/2^)
MDT TMR 2103 [23]	6	∼10
Magic 2001 [24]	5	—
This work	9.7	0.79

The bonded double‐layer TMR device fabricated in this study exhibited the lowest magnetic noise at 1 Hz, reaching the hundreds of pT·Hz^−1/2^ level. In contrast, the noise levels of commercial TMR sensors were typically several nT·Hz^−1/2^ under comparable operating conditions. In addition, the magnetic sensitivity of the proposed device was higher than that of the commercial devices. These results collectively demonstrate that the 3D bonded TMR architecture effectively enhances both low‐frequency noise suppression and magnetic field sensitivity, outperforming existing high‐performance TMR sensor chips currently available on the market.

## 4 Conclusion

In this study, we addressed the critical challenges associated with conventional lateral scaling of MTJ array fabrication, including increased parasitic resistance, elevated process complexity, and difficulty in suppressing low‐frequency noise. We proposed and experimentally validated a novel TMR device scheme based on 3D heterogeneous integration and bonding. Our approach achieves wafer‐level vertical bonding of double‐layer TMR films and overcomes the limitations of planar dimensions and effectively doubles the number of series‐connected MTJs.

At the process level, the sputtering parameters, argon ion activation conditions, and bonding pressure were systematically optimized to minimize the bonding interface roughness to 0.509 nm and achieve a void ratio of only 0.73%. Thus, the magnetic properties and tunneling effect of the films were preserved. Furthermore, when combined with an optimized etching angle of 45°, sidewall redeposition was effectively mitigated, ensuring proper operation of both the upper and lower MTJs.

Experimental results demonstrated significant performance improvements: under an etching angle of 45°, the device resistance reached 1.9 times that before bonding, the MR ratio increased from 149% to 172%, and the low‐frequency 1/f magnetic noise at 1 Hz decreased from 1.92 nT to 0.97 nT, achieving nearly 50% suppression.

In summary, this work not only addresses process‐level challenges, such as interface roughness, bonding reliability, and sidewall redeposition in wafer‐bonded TMR arrays, but also realizes the synergistic optimization of noise suppression and MR enhancement at the device level. The proposed 3D heterogeneous integration and bonding technique provides a practical and scalable approach for fabricating high‐density, low‐noise, low parasitic resistance TMR sensor arrays and has application potential and broad prospects for future development.

## 5 Statistical Analysis

Statistical analyses were performed to ensure the reliability and reproducibility of the measured device performance.

1) Data pre‐processing. Raw magnetic measurement data were first inspected to remove evident recording errors.

2) Data presentation. All results were reported as mean ± SD unless otherwise specified. The distribution plots (surface roughness, bonding yield, MR ratio, 1/f noise, and parasitic resistance) show the mean values for clarity.

3) Sample size. For each statistical comparison, the sample size n is indicated in the corresponding figure legend. Typically, n = 15 devices per group were used for MR, noise, and sensitivity measurements, and n = 15 wafers were used for bonding yield and uniformity analyses.

4) Software. Statistical analyses were conducted using OriginPro 2025.

## Funding

This work was supported in part by the National Key Research and Development Program of China (2023YFB2407800), National Natural Science Foundation of China (grant no. 62271469), Beijing Municipal Natural Science Foundation (grant no. L255006), Science and Disruptive Technology Program, AIRCAS (2025‐AIRCAS‐SDTP‐02), Young Elite Scientists Sponsorship Program by CAST (no. YESS20210341), the One Hundred Person Project of the Chinese Academy of Sciences, and the Xiaomi Young Talents Program.

## Conflicts of Interest

The authors declare no conflicts of interest.

## Data Availability

The data that support the findings of this study are available from the corresponding author upon reasonable request.
