# Correction to “Three‐Dimensional Heterogeneous Bonding for High‐Density and Low‐Noise TMR Sensing Arrays”

**DOI:** 10.1002/advs.76259

**Published:** 2026-06-23

Han Z, Jin Z, Zhang C, Chen J. “Three‐Dimensional Heterogeneous Bonding for High‐Density and Low‐Noise TMR Sensing Arrays,” *Advanced Science*, 13 no. 13 (2026): e20686. https://doi.org/10.1002/advs.202520686

On page 5 of the published article, under section 3.3 “Magnetic Characterization of Films viaVibrating Sample Magnetometry,” the coercive field values “1.43 × 10^−4^ Oe and 2.46 × 10^−4^ Oe” were incorrect. This should have read: “1.43 Oe and 2.46 Oe.” This correction does not affect the results, discussion, or conclusions of the article.

We apologize for this error.

